# Endothelin-1 induces LIMK2-mediated programmed necrotic neuronal death independent of NOS activity

**DOI:** 10.1186/s13041-015-0149-3

**Published:** 2015-10-06

**Authors:** Ah-Reum Ko, Hye-Won Hyun, Su-Ji Min, Ji-Eun Kim, Tae-Cheon Kang

**Affiliations:** Department of Anatomy & Neurobiology, Institute of Epilepsy Research, College of Medicine, Hallym University, Chunchon, Kangwon-Do 200-702 South Korea

**Keywords:** DRP1, Endothelin-1, ET_B_ receptor, LIMK2, Neuronal death, NOS, Mitochondrial fission, Sezuire, Status epilepticus, ROCK1

## Abstract

**Background:**

Recently, we have reported that LIM kinase 2 (LIMK2) involves programmed necrotic neuronal deaths induced by aberrant cyclin D1 expression following status epilepticus (SE). Up-regulation of LIMK2 expression induces neuronal necrosis by impairment of dynamin-related protein 1 (DRP1)-mediated mitochondrial fission. However, we could not elucidate the upstream effecter for LIMK2-mediated neuronal death. Thus, we investigated the role of endothelin-1 (ET-1) in LIMK2-mediated neuronal necrosis, since ET-1 involves neuronal death via various pathways.

**Results:**

Following SE, ET-1 concentration and its mRNA were significantly increased in the hippocampus with up-regulation of ET_B_ receptor expression. BQ788 (an ET_B_ receptor antagonist) effectively attenuated SE-induced neuronal damage as well as reduction in LIMK2 mRNA/protein expression. In addition, BQ788 alleviated up-regulation of Rho kinase 1 (ROCK1) expression and impairment of DRP1-mediated mitochondrial fission in CA1 neurons following SE. BQ788 also attenuated neuronal death and up-regulation of LIMK2 expression induced by exogenous ET-1 injection.

**Conclusion:**

These findings suggest that ET-1 may be one of the upstream effectors for programmed neuronal necrosis through abnormal LIMK2 over-expression by ROCK1.

## Background

Necrosis and apoptosis are two major cell death patterns: Necrosis is a passive cell death, while apoptosis is a highly controlled process [1, 2]. Interestingly, some necrotic processes can be mediated by receptor interacting protein kinase 1 (RIP1), which is termed programmed necrosis or necroptosis [3–6]. Recently, we have reported that aberrant cyclin D1 expression induced by up-regulation of LIMK2 (one of F-actin regulators) expression evokes programmed necrotic neuronal death following SE (prolonged seizure activity, [7]). Briefly, SE down-regulates p27^Kip1^ expression by ROCK activation, which induces cyclin D1/cyclin dependent kinase 4 (CDK4) expressions in neurons vulnerable to SE, and subsequently increases LIMK2 expression independent of RIP1 and caspase-3 activity. In turn, up-regulated LIMK2 impairs DRP1-mediated mitochondrial fission that finally provokes programmed necrotic death. Indeed, LIMK2 knockdown and rescue of mitochondrial fission attenuates this programmed necrotic neuronal death. However, we could not elucidate the upstream effecter for LIMK2-mediated programmed necrotic neuronal death.

ET-1 is one of vasoactive peptides that may be responsible for maintaining the tone of the cerebral vasculature. ET-1 exerts various actions by binding to two specific G-protein-coupled receptors subtypes, ET_A_ and ET_B_ receptors. ET_B_ receptors predominantly express in the brain parenchyma. In contrast, ET_A_ receptors localize in vascular smooth muscle within the brain parenchyma [8]. ET_B_ receptor activations elevate intracellular Ca^2+^ concentration in cultured neurons and hippocampal slices in an autocrine-signaling mode [9–11]. This intracellular mobilization of Ca^2+^ rapidly leads to Ca^2+^-dependent NO synthesis. NO reacts with superoxide anion to form peroxynitrite anion (ONOO^−^), which is a highly reactive oxidizing agent capable of causing tissue damage [12] and regulating mitochondrial length [13]. ET_B_ receptors activations also stimulate cyclin D1 expression, which coordinates mitochondrial bioenergetics and provokes dysfunction of mitochondrial fission [7, 14, 15]. These events all participate in the neuronal damage in various neurological diseases. Indeed, exogenous ET-1 injection into the brain parenchyma results in pan-necrosis [16]. Therefore, it is likely that ET-1 may involve LIMK2-mediated impairment of mitochondrial dynamics during neuronal death in ET_B_ receptor-mediated NOS activation-independent or -dependent manner.

To elucidate this hypothesis, we investigated whether ET-1 is involved in LIMK2-mediated neuronal death. Here, we describe a novel action of ET-1 in LIMK2-mediated neuronal death. Following SE, ET-1 up-regulated ROCK1 and LIMK2 expressions in neurons vulnerable to SE via ET_B_ receptor activation independent of NO production. In addition, exogenous ET-1 injection impaired mitochondrial fission resulting LIMK2-mediated neuronal necrosis. Therefore, our findings suggest that ET-1 may be one of the inducing factors for LIMK2-mediated programmed necrosis following SE.

## Results

### SE rapidly releases ET-1 and induces ET_B_ receptor expression in neurons

**Fig. 1:**
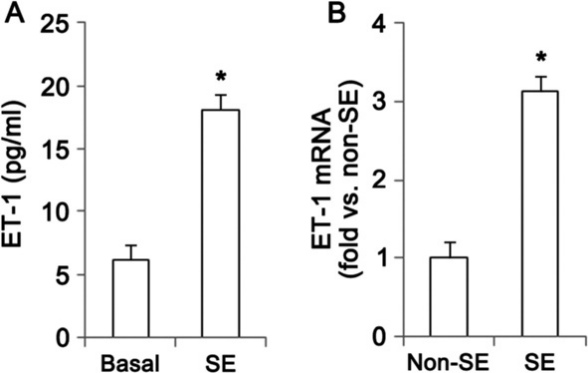
SE-induced alterations in ET-1 mRNA/protein expressions and its release at 4 h after SE. **a** Quantitative values (mean ± S.E.M) of big ET-1 concentration (*n* = 10 per each group). Significant differences from non-SE animals, *p < 0.05. **b** Quantitative values (mean ± S.E.M) of ET-1 mRNA in the hippocampus (*n* = 10 per each group). Significant differences from non-SE animals, *p < 0.05

**Fig. 2:**
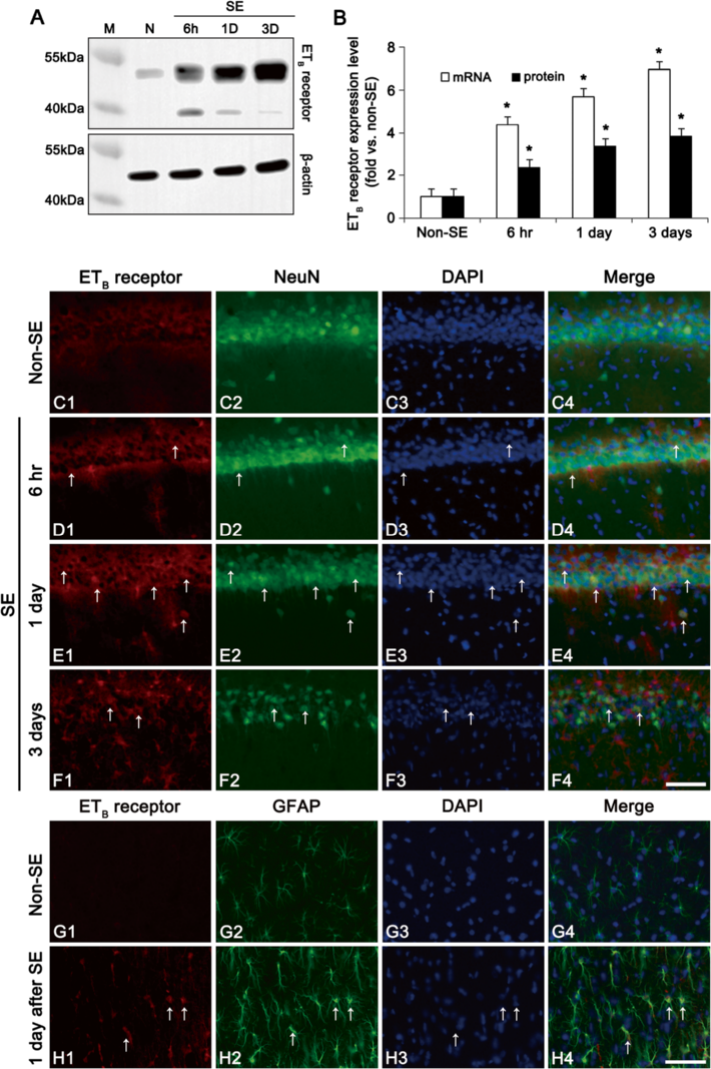
Protein expression levels and cellular localization of ET_B_ receptor following SE. **a** Western blot image of ET_B_ receptor in the hippocampus. ET_B_ receptor expression is gradually increased at 6 h – 3 days after SE. **b** Quantitative values (mean ± S.E.M) of ET_B_ receptor expression level in the hippocampus, based on western blot (*n* = 10 per each group). Significant differences from non-SE animals, *p < 0.05. **c**-**f** Representative photographs of ET_B_ receptor and NeuN in the CA1. Following SE, ET_B_ receptor expression is gradually elevated in CA1 pyramidal cells. Three days after SE, NeuN immunostains show neuronal damage. Arrows indicate neurons containing ET_B_ receptor expression. Bar = 50 μm. **g**-**h** Representative photographs of ET_B_ receptor and GFAP. ET_B_ receptor expression is weakly observed in a few astrocyte in non-SE animals. One day after SE, ET_B_ receptor expre**s**sion is elevated in astrocyte. Arrows indicate astrocytes containing ET_B_ receptor expression. Bar = 50 μm

In the present study, microdialysis analysis revealed that big ET-1 concentration in the hippocampus was 6.1 ± 0.9 pg/ml in basal condition. Big ET-1 concentration was elevated to 18.1 ± 3.9 pg/ml at 4 h after SE (Fig. 1). ET-1 mRNA in the hippocampus was also increased to 3.12-fold of non-SE animals following SE (Fig. 1). ET_B_ receptor expression was weakly detected in a few CA1 pyramidal neurons of non-SE animals (Fig. 2a-c). Six hr – 1 day post-SE animals, ET_B_ receptor expression was markedly elevated in CA1 pyramidal cells (Fig. 2a-e, *p* < 0.05 vs. non-SE animals). In this time point, ET_B_ receptor expression was also elevated in astrocytes (Fig. 2e, g and h, *p* < 0.05 vs. non-SE animals). Three days after SE, ET_B_ receptor expression was significantly reduced in CA1 neurons due to massive neuronal loss, while its expression was enhanced in astrocytes (Fig. 2f). These findings indicate that SE may increase ET-1 synthesis and up-regulate ET_B_ receptor expression in neurons as well as astrocytes.

### ET_B_ receptor activation induces neuronal death in NOS-independent pathway following SE

**Fig. 3:**
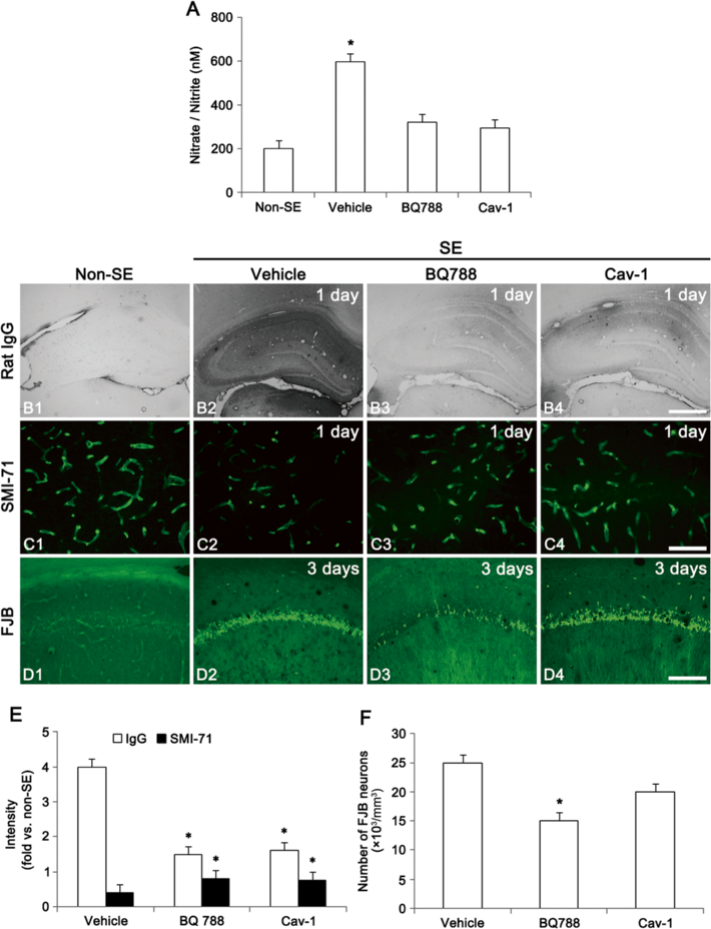
Effect of BQ788 and Cav-1 peptide on vasogenic edema, BBB and neuronal damage following SE. **a** Quantitative values (mean ± S.E.M) of nitrate/nitrite (NO products) levels in the hippocampus at 4 h after SE (*n* = 10 per each group). Significant differences from non-SE animals, *p < 0.05. **b** Representative photographs of vasogenic edema formation at 1 day after SE. Both BQ788 and Cav-1 peptide treatments attenuate SE-induced vasogenic edema. Bar = 400 μm. **c** Representative photographs of SMI-71 in the CA1 at 1 day after SE. In non-SE animals, SMI-71 expression is detected in most of vessels. Following SE, SMI-71 immunoreactivity is decreased. Both BQ788 and Cav-1 peptide treatments attenuate SE-induced BBB breakdown. Bar = 50 μm. **d** FJB-positive neuronal damage in the CA1 at 3 days after SE. BQ788 infusion attenuates SE-induced neuronal damage, while Cav-1 peptide infusion does not affect SE-induced neuronal damage. Bar = 100 μm. **e** Quantitative values (mean ± S.E.M) of IgG and SMI-71 expression in the hippocampus (*n* = 10 per each group). Significant differences from vehicle, *p < 0.05. **f** Quantitative values (mean ± S.E.M) of FJB-positive degenerating neurons (*n* = 10 per each group). Significant differences from vehicle, *p < 0.05

Since ET-1 triggers signaling cascades for the production of NO [17], we confirmed whether ET-1-mediated NO production involves in neuronal damage induced by SE. The present data demonstrated that NO product level was increased from 213.9 ± 81.1 to 547.6 ± 94.9 nM at 4 h after SE (Fig. 3a). Consistent with our previous study [18], vasogenic edema and reduction in SMI-71 (a BBB marker) were detected in the hippocampus 1 day after SE (*p* < 0.05 vs. non-SE animals, Fig. 3b, c and e). Both BQ788 and Cav-1 peptide (a NOS inhibitor) treatments effectively attenuated vasogenic edema and BBB breakdown induced by SE (*p* < 0.05 vs. vehicle, Fig. 3a-c and e). However, Cav-1 peptide infusion did not affect SE-induced neuronal damage, while BQ788 infusion attenuated it at 3 days after SE (*p* < 0.05 vs. vehicle, Fig. 3d and f). These findings indicate that ET-1 may be involved in neuronal death via ET_B_ receptor-mediated pathways independent of NOS following SE.

### Blockade of ET_B_ receptor function prevents SE-mediated LIMK2 induction

**Fig. 4:**
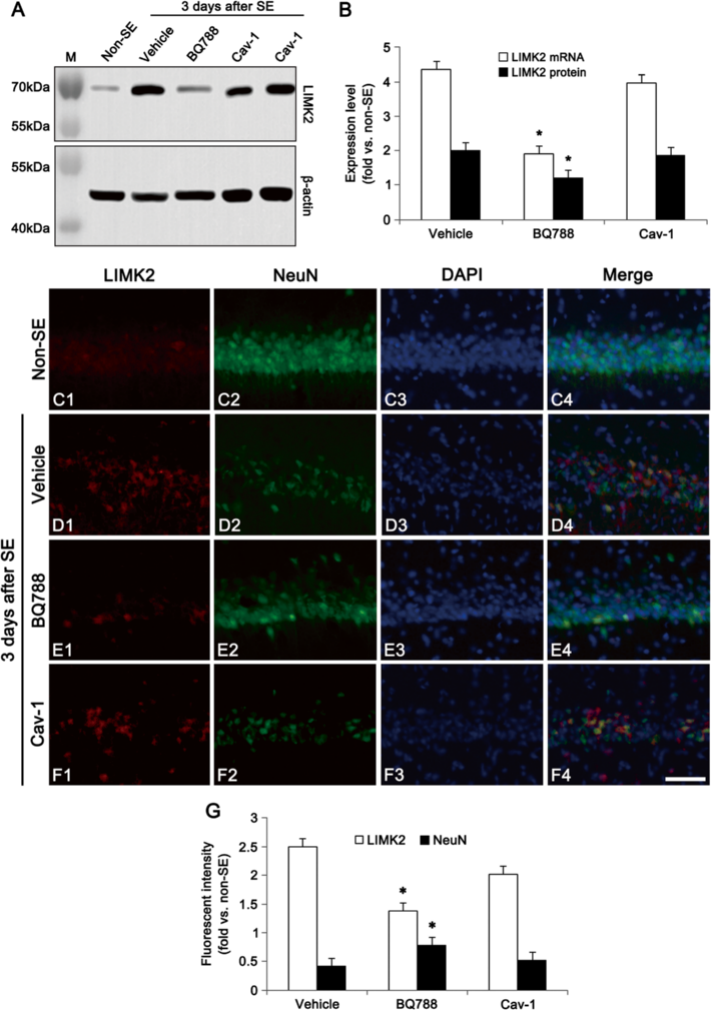
Effect of BQ788 and Cav-1 peptide on SE-induced LIMK2 expression at 3 days after SE. **a** Western blot image of LIMK2 in the hippocampus. BQ-788 infusion attenuates up-regulation of LIMK2 expression induced by SE, while Cav-1 peptide does not. **b** Quantitative values (mean ± S.E.M) of LIMK2 mRNA/protein expression level in the hippocampus (*n* = 10 per each group). Significant differences from vehicle, *p < 0.05. **c-f** Representative photographs of LIMK2 and NeuN in the CA1 pyramidal cells. As compared to vehicle, BQ788 inhibits LIMK2 induction following SE. Bar = 50 μm. **g** Quantitative values (mean ± S.E.M) of LIMK2 and NeuN in the hippocampus (*n* = 10 per each group). Significant differences from vehicle, *p < 0.05

Next, we tested whether ET_B_ receptor activity influences SE-induced LIMK2 induction. Similar to our previous study [7], western blot study showed the up-regulation of LIMK2 expression at 3 days after SE (*p* < 0.05 vs. non-SE animals, Fig. 4a and b). LIMK2 mRNA was also increased to 4.35-fold of the non-SE level in this time point (*p* < 0.05, Fig. 4d). BQ788 infusion effectively inhibited up-regulation of LIMK2 mRNA/protein expression at 3 days after SE (*p* < 0.05 vs. vehicle, Fig. 4a and b). However, Cav-1 peptide treatment did not affect LIMK2 mRNA/protein expression in this time point (Fig. 4a and b). Immunofluorescence data also showed up-regulated LIMK2 expression in CA1 pyramidal cells following SE (*p* < 0.05 vs. non-SE), and only BQ788 attenuated this up-regulation of LIMK2 expression induced by SE (*p* < 0.05 vs. vehicle, Fig. 4c-g). Taken together, the present data indicate that ET_B_ receptor activation may play an important role in SE-induced LIMK2 induction independent of NO productions.

### ET_B_ receptor-mediated ROCK1 expression induces neuronal death following SE

**Fig. 5:**
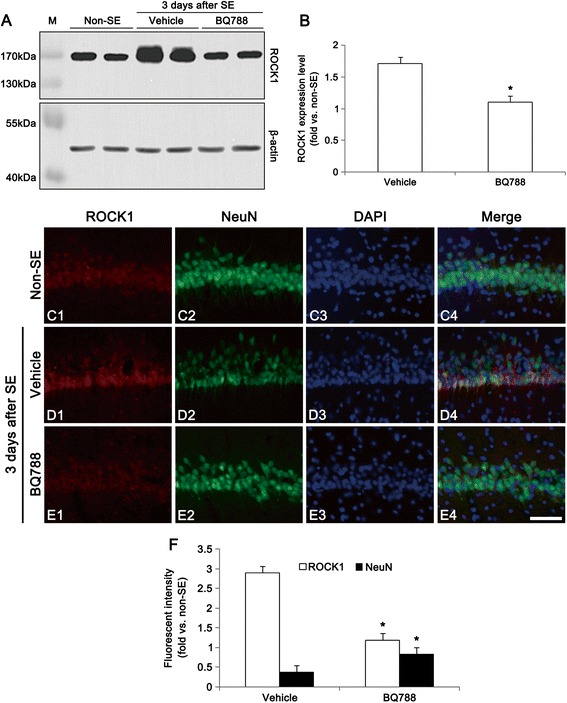
Effect of BQ788 and Cav-1 peptide on SE-induced ROCK1 expression at 3 days after SE. **a** Western blot images of ROCK1 in the hippocampus. BQ-788 infusion attenuates up-regulation of ROCK1 expression induced by SE. **b** Quantitative values (mean ± S.E.M) of ROCK1 expression level in the hippocampus (*n* = 10 per each group). Significant differences from vehicle, *p < 0.05. **c-e** Representative photographs of ROCK1 and NeuN in the CA1 pyramidal cells. As compared to non-SE, ROCK1 expression is increased and NeuN expression is decreased at three days after SE. BQ788 attenuates up-regulation of ROCK1 expression and down-regulation of NeuN expression by SE. Bar = 50 μm. **f** Quantitative values (mean ± S.E.M) of ROCK1 and NeuN in the hippocampus, based on immunofluorescent study (*n* = 10 per each group). Significant differences from vehicle, *p < 0.05

Since ROCK involves SE-induced neuronal death by LIMK2 induction [7], we validated the effect of BQ788 on ROCK1 expression in SE-induced neuronal death. Three days after SE, up-regulated ROCK1 expression was observed in CA1 pyramidal cells (*p* < 0.05 vs. non-SE, Fig. 5), which was alleviated by BQ788 infusion (*p* < 0.05 vs. vehicle, Fig. 5). These findings indicate that ET_B_ receptor activation may result in ROCK1-mediated LIMK2 induction following SE.

**Fig. 6:**
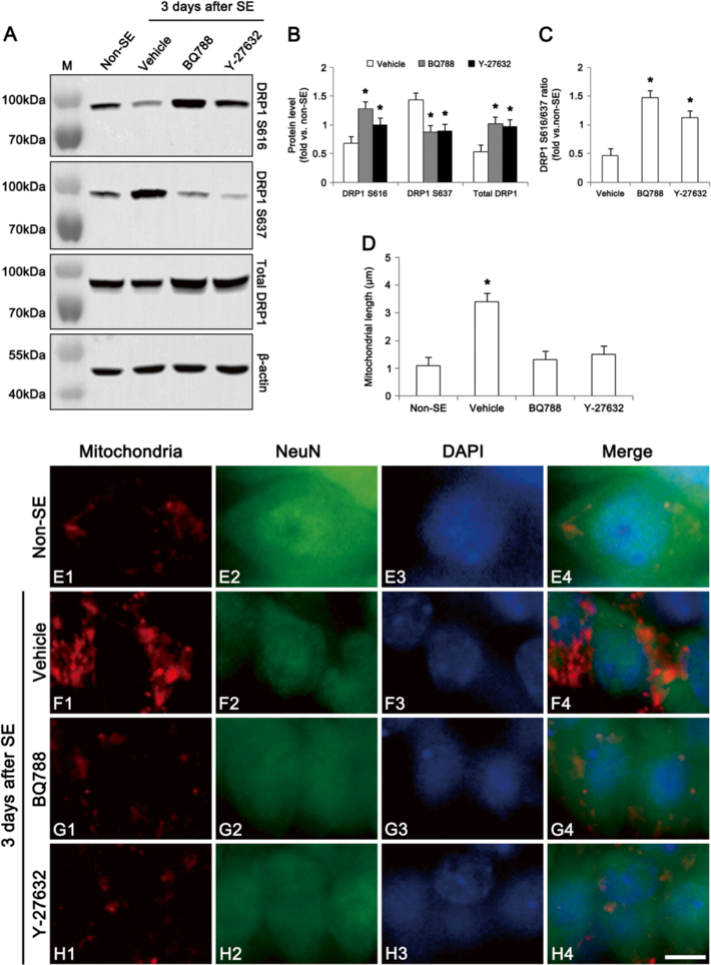
Effect of BQ788 and Y-27632 on dysfunction of mitochondrial fission at 3 days after SE. **a** Western blot images of DRP1, DRP1 S616 and DRP1 S637 in the hippocampus. As compared to vehicle, both BQ788 and Y-27632 (a ROCK inhibitor) attenuate the reductions in DRP1 and DRP1 S616 expression, but increase DRP1 S637 expression. **b** Quantitative values (mean ± S.E.M) of DRP1, DRP1 S616, DRP1 S637 level (*n* = 10 per each group). Significant differences from vehicle, *p < 0.05. **c** Quantitative values (mean ± S.E.M) of DRP1 S616/S637 ratio in the hippocampus (*n* = 10 per each group). Significant differences from vehicle, *p < 0.05. **d** Quantitative values (mean ± S.E.M) of mitochondrial length in the CA1 neurons (*n* = 10 per each group). Significant differences from non-SE animals, *p < 0.05 **e-h** Representative photographs of mitochondria and NeuN in the CA1 neurons. SE increases mitochondrial length and sphere formation. Both BQ788 and Y-27632 alleviate mitochondrial elongation and sphere formation induced by SE. Bar = 6.25 μm

Recently, we have reported that impairment of LIMK2-mediated mitochondrial dynamics may participate in the neuronal necrosis following SE [7]. Since DRP1 S616 phosphorylation accelerates mitochondrial fission, but S637 phosphorylation increase the detachment of DRP1 from mitochondria resulting in inhibition of mitochondrial fission [19], we investigated whether ET_B_ receptor activation is related to impairment of mitochondrial dynamics following SE. Consistent with our previous study [7], SE reduced DRP1 expression and DRP1 S616/S637 phosphorylation ratio (Fig. 6a-c), but induced mitochondrial elongation and sphere formation in CA1 neurons (*p* < 0.05 vs. non-SE animals, Fig. 6d-h). Both BQ788 and Y-27632 (a ROCK inhibitor) attenuated the reductions in DRP1 S616/S637 phosphorylation ratio and DRP1 expression (*p* < 0.05 vs. vehicle, Fig. 6a-c), and inhibited mitochondrial elongation and sphere formation following SE (*p* < 0.05 vs. vehicle, Fig. 6d-h). These findings indicate that ET_B_ receptor activation may involve LIMK2-DRP1-mediated impairment of mitochondrial fission during programmed necrotic cell death.

### Exogenous ET-1 injection induces LIMK2-mediated neuronal death in the hippocampus

**Fig. 7:**
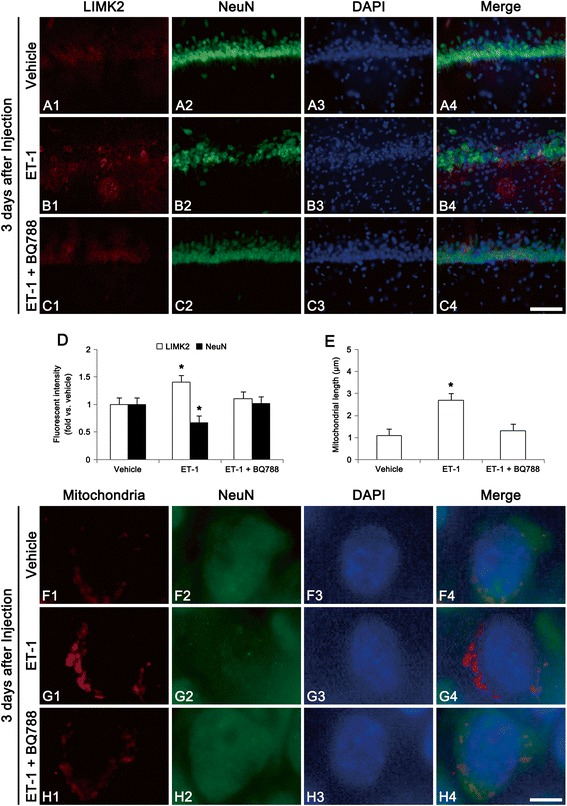
Effect of exogenous ET-1 on LIMK2 expression and mitochondrial fission at 3 days after injection. **a-c** Representative photographs of LIMK2 and NeuN in the CA1. As compared to vehicle, ET-1 injection increases LIMK2 expression accompanied by reduction in NeuN expression. Co-treatment of ET-1 and BQ788 attenuates up-regulation of LIMK2 expression induced by ET-1. Bar = 100 μm. **d** Quantitative values (mean ± S.E.M) of LIMK2 and NeuN (*n* = 10 per each group). Significant differences from vehicle, *p < 0.05. **e** Quantitative values (mean ± S.E.M) of mitochondrial length (*n* = 10 per each group). Significant differences from vehicle, *p < 0.05. **f-h** Representative photographs of mitochondria and NeuN in the CA1 neurons. ET-1 injection increases mitochondrial length and sphere formation, as compared to vehicle. Co-treatment of ET-1 and BQ788 prevents mitochondrial elongation and sphere formation induced by ET-1. Bar = 6.25 μm

To investigate the direct role of ET-1 in LIMK2-mediated neuronal death, we injected ET-1 into the hippocampus of normal rats. As compared to vehicle, ET-1 (40 pmol/μl) increased neuronal LIMK2 expression, accompanied by reduction in NeuN expression at 3 days after injection (*p* < 0.05, Fig. 7a, b and d). Co-treatment of ET-1 and BQ788 attenuated up-regulation of LIMK2 expression induced by ET-1 in this time point (*p* < 0.05 vs. vehicle, Fig. 7c and d). ET-1 injection also induced mitochondrial elongation and sphere formation, as compared to vehicle (*p* < 0.05, Fig. 7e-g). Co-treatment of ET-1 and BQ788 prevented mitochondrial elongation and sphere formation induced by ET-1 (*p* < 0.05 vs. vehicle, Fig. 7e and h). These findings also support that ET_B_ receptor activation may play an important role in LIMK2-mediated impairment of mitochondrial dynamics during programmed necrotic cell death.

## Discussion

The increases in the production and release of ET-1 are involved in the various pathological response of the brain other than vascular constriction [20–25]. Indeed, ET_B_ receptor activation plays a substantial role as a proliferative and anti-apoptotic factor [26–28]. However, ET-1 also evokes necrotic neuronal damage [13], and causes reactive nitrogen species-mediated tissue injury [12]. In the present study, ET_B_ receptor expression was markedly elevated in CA1 pyramidal cells and astrocytes following SE, accompanied by the rapid release of ET-1. The present study also demonstrates that both BQ788 and Cav-1 peptide effectively inhibited SE-induced vasogenic edema and BBB breakdown. Therefore, it would be likely that ET_B_ receptor-mediated NOS activation might affect neuronal death via vasogenic edema formation or excessive reactive oxidizing production following SE. However, BQ788 infusion attenuated SE-induced neuronal damage, while Cav-1 peptide infusion did not affect it. Therefore, these findings indicate that ET-1 may participate in neuronal death via ET_B_ receptor-mediated pathways following SE, which may be vasogenic edema- and NOS-independent mechanism.

LIMK2 regulates cofilin activity, which is one of the regulators of actin dynamics. Interestingly, LIMK2 also modulates cyclin D1 repression [7, 29]. Recently, we have reported that SE increases LIMK2 expression and dysfunctions DRP1-mediated mitochondrial fission in necrotic neurons, and LIMK2 knockdown attenuates necrotic neuronal damage by recovery of impaired mitochondrial fission [7]. Consistent with this previous study, the present data show that SE up-regulated LIMK2 expression in CA1 neuronal vulnerable to SE, which was accompanied by impairment of DRP1-mediated mitochondrial fission. Furthermore, exogenous ET-1 injection resulted in LIMK2 over-expression and dysfunction of mitochondrial fission. In addition, BQ788 significantly inhibited SE- and exogenous ET-1-induced LIMK2 expression. These findings indicate that ET_B_ receptor activation may play an important role in SE-induced LIMK2 induction and dysfunction of mitochondrial fission independent of NO productions. A dysfunction of mitochondrial fission improperly segregates mitochondria, which decreases ATP levels [19, 30]. Furthermore, elongated mitochondria cannot be transported to proper distal regions in either dendrites or axons resulting the local limit of ATP supply [31, 32]. DRP1 deletion also inhibits mitochondrial respiratory function and increases the reactive oxygen species production [33, 34]. Following DNA damage, DRP1 overexpression increases neuronal viability by restoring the mitochondrial dynamics [35]. Since DRP1 is required for caspase activation during apoptosis [36], it is likely that LIMK2-mediated reduction in DRP1 expression may prefer to inducing necrosis rather than apoptosis. Therefore, these findings suggest that ET-1 may be involve in neuronal necrosis by up-regulation of LIMK2, which provokes impairment of DRP1-mediated mitochondrial dynamics.

Although the underlying mechanism is still unknown, ROCK inhibitors have neuroprotective effects from various neuronal injuries [37, 38]. Recently, we have reported that ROCK inhibitor down-regulates LIMK2 expression by up-regulation of p27^Kip1^ expression following SE [7]. Furthermore, ROCK is one of the effectors for ET-1 mediated signaling pathway [39–41]. The present study demonstrates that DRP1 expression, DRP1 S616/S637 phosphorylation ratio and mitochondrial fission were reduced with ROCK1 over-expression following SE, which were inhibited by both BQ788 and Y-27632. These findings demonstrate that ROCK1-induced LIMK2 over-expression may be the novel underlying mechanism for ET-1-induced neuronal death.

ET_B_ receptor activation leads to severe vasogenic edema via the impairment of aquaporin-4 (AQP4, a water channel) in astrocytes within the piriform cortex (PC) following SE [42]. In the present study, up-regulation of ET_B_ receptor expression was observed in astrocytes and CA1 neurons. Furthermore, BQ788 infusion effectively prevented SE-induced vasogenic edema formation as well as neuronal death in the hippocampus. Based on the inhibitory role of ET-1 in astroglial AQP4 functionality [42], these findings suggest that up-regulated ET_B_ receptor expression in astrocytes may involve the dysfunction of AQP4 in astrocytes and lead to vasogenic edema in the hippocampus, like the PC.

## Conclusion

**Fig. 8:**
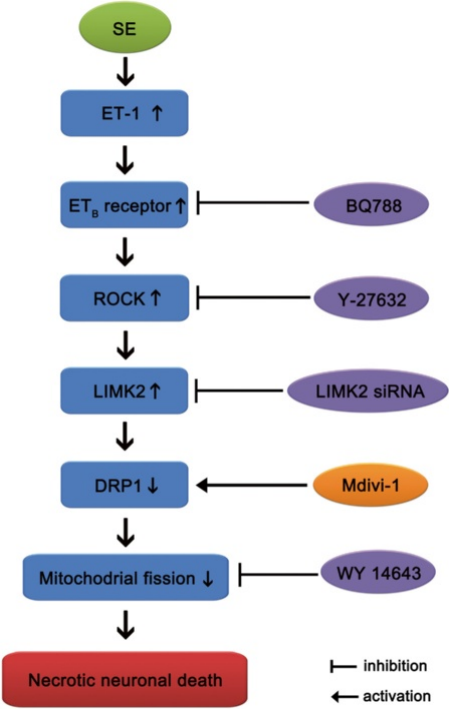
Scheme of programmed necrotic neuronal death based on the present data and a previous report [7]. Increased ET-1 release and ET_B_receptor expression induced by SE results in the up-regulation of ROCK1 expression, which subsequently increases LIMK2 expression independent of NOS activity. The up-regulated LIMK2 induces necrotic neuronal death by impairment of DRP1-mediated mitochondrial fission. Inhibition of LIMK2 expression and rescue of DRP1 function attenuate this programmed necrotic neuronal death

In summary, ET-1-mediated signal is involved in mitochondrial dynamics during neuronal necrosis (Fig. 8). These findings suggest that ET-1 may be involved in SE-induced neuronal necrosis independent of NOS synthesis and BBB disruption. Therefore, ET-1-mediated signaling pathway may be an important therapeutic target for programmed necrotic neuronal death.

## Methods

### Experimental animals and chemicals

Male Sprague–Dawley (SD) rats were obtained from Experimental Animal Center, Hallym University, Chunchon, South Korea, and and housed in standard rodent cages (3 rats per cage) at 22 ± 2 °C, 55 ± 5 % humidity and a 12:12 light/dark cycle. Animals had free access to food and water. After at least 1 week of adaptation in the animal. Experimental procedures were done on approval of the Institutional Animal Care and Use Committee of the Hallym university (Chunchon, Republic of Korea). All reagents were obtained from Sigma-Aldrich (St. Louis, MO, USA), unless otherwise noted.

### Surgery

For microdialysis and ET-1 injection, rats were anesthetized with 1–2 % Isoflurane in O_2,_ and placed in a stereotaxic frame. A microdialysis guide cannula was inserted in the right hippocampus using the following coordinates: 3 mm posterior; 2 mm lateral; 3.2 mm depth from bregma [43]. Seven days after surgery, animals were used for microdialysis. Some animals were inserted with a cannula (27 G) by the same methods. ET-1 (40 pmol in 1 μl of saline) or mixture of ET-1 (80 pmol in 0.5 μl of saline) and BQ-788 (an ET_B_ receptor antagonist; 6 pmol in 0.5 μl of saline) was infused over a 5-min period using a microinjection pump (0.2 μl/min, KD scientific, Hollistone, MA, USA). For control, rats were given 1 μl of saline instead of ET-1. Three days after injection, we used them for immunohistochemical study.

Other animals were implanted with a brain infusion kit 1 (Alzet, USA) into the right lateral ventricle using the following coordinates: 1 mm posterior; 1.5 mm lateral; −3.5 mm depth. The implanted osmotic pump (1007D, Alzet, USA) was connected over 7 days that continuously infused (1) vehicle, (2) BQ-788 (3 pmol), (3) Cav-1 peptide (a NOS inhibitor; 5 μM), and (4) Y-27632 (a ROCK1 inhibitor; 10 μM) into the ventricle. In the preliminary and our previous studies [42, 44], seizure severity and BBB integrity were unaffected by each compound infusion [45–47].

### Seizure induction

Three days after surgery, SE was induced by a systemic injection of pilocarpine (380 mg/kg, I.P.). To reduce peripheral effects of pilocarpine, Atropine methylbromide (5 mg/kg, I.P.) was injected 20 min before a single dose of pilocarpine. Animals were maintained in SE for 2 h, after which diazepam (10 mg/kg, i.p.) was administered to terminate seizure activity, and repeated, as needed. As controls, age-matched normal rats were treated with saline instead of pilocarpine.

### ET-1 and NO assay

One day before SE induction, a microdialysis probe (CMA 12) was inserted into the hippocampus. The microdialysis probe was perfused with Ringer’s solution [42]. The perfusion rate was 1 μl/min for 4 h before/after SE induction, and efflux from the microdialysis probe was collected 240 μl, respectively. To measure ET-1 and NO concentrations in perfusates, we used ET-1 ELISA kit (Enzo Life Science) and nitrate/nitrite assay kit (Cayman chemical company, USA), according to the manufacturer’s instructions [42].

### Tissue processing

Rat were transcardially perfused with phosphate-buffered saline (PBS) followed by 4 % paraformaldehyde in phosphate buffer (PB, 0.1 M, pH 7.4) [42]. Brains were removed and post-fixed in the same fixative for 4 h, then moved to 30 % sucrose solution until saturated and then frozen and sectioned at 30 μm on a cryostat. Consecutive sections were contained in six-well plates containing PBS [48]. For western blot, the hippocampal protein was obtained by being homogenized and centrifugated [42], then the supernatant was collected. The total protein concentration was assayed by a Micro BCA Protein Assay Kit (Pierce Chemical, Rockford, IL, USA). For quantitative real-time PCR, total RNA in the hippocampus was obtained using Trizol Reagents, according to the manufacturer’s protocol (Ambion, Austin, TX, USA) [42].

### Immunohistochemistry

To measure vasogenic edema lesion, tissue sections were immersed for 10 min in 3 % H_2_O_2_ and 30 min in blocking solution (10 % normal horse serum in PBS). Horse anti-rat IgG (Vector, USA) was applied overnight at 4 °C. Immunoreactivity was developed with 3,3′-diaminobenzidine. We analyzed the volume of vasogenic edema lesion with the modified Cavalieri method [18, 49]. Table 1 is a list of the antibodies used in double immunofluorescent study. Sections were incubated in a mixture of antisera overnight at room temperature, and subsequently in a mixture of FITC- and Cy3-conjugated IgG (Jackson Immunoresearch Laboratories Inc., West Grove, PA, USA; diluted 1:250). To verify the specificity of the antibodies (negative controls), a primary antibody was omitted. Images were taken with an AxioImage M2 microscope. Fluorescent intensity was measured using AxioVision Rel. 4.8 software and ImageTool program V. 3.0. [42, 50].

**Table 1:** Primary antibodies used in the present study

Antibody	Host	Manufacturer (catalog number)	Dilution used
DRP1	Rabbit	Thermo (PA1-16987)	1:1000 (WB)
DRP1 S616	Rabbit	Cell signalling (#4867)	1:1000 (WB)
DRP1 S637	Rabbit	Cell signalling (#4494)	1:1000 (WB)
Endothelin 1 (ET-1)	Rabbit	Abbiotec (250633)	1:200 (IF)
Endothelin B receptor (ET_B_ receptor)	Rabbit	Millipore (AB3284)	1:50 (IF)
			1:1000 (WB)
Glial fibrillary acidic protein (GFAP)	Mouse	Chemicon (MAB3402)	1:4000 (IF)
LIMK2	Rabbit	Abcam (ab45165)	1:100 (IF)
			1:2000 (WB)
Mitochondrial marker	Mouse	Millipore (MAB3494)	1:50 (IF)
NeuN	Mouse	Millipore (MAB377)	1:500 (IF)
ROCK1	Rabbit	Abcam (ab45171)	1:100 (IF)
			1:1000 (WB)
SMI-71	Mouse	Covance (SMI-71R)	1:1000 (IF)

IF, Immunofluorescence; WB, Western blot

### Fluoro-Jade B staining

Sections mounted on gelatin-coated slides were immersed in in 80 % ethanol containing 1 % sodium hydroxide. Then tissue sections were immersed in 70 % ethanol for 2 min and distilled water for 2 min. After immersion in potassium permanganate for 15 min, tissues were washed with distilled water and then incubated in 0.001 % FJB (Histo-Chem Inc. Jefferson, AR, USA). Next, the slides were rinsed, dehydrated, and finally mounted with DPX. Two different investigators performed cell counts with optical dissector methods [7].

### Western blot

Equal quality protein (20 μg) of every sample were loaded into 10 % polyacrylamide gel before transferring to nitrocellulose transfer membranes (Schleicher and Schuell BioScience Inc.). Membranes were blocked with 5 % non-fat dry milk, incubated with the primary antibody (Table 1), and processed for visualization [7]. All results were normalized against β-Actin (1:2000; Sigma) [51].

### Quantitative real-time PCR

Quantitative real-time PCR was performed using the MyiQ Single Color Real-Time PCR System (Bioneer, Taejon, South Korea). The primers used in the present study were as follows: Forward GACCAGCGTCCTTGTTCCAA, Reverse TTGCTACCAGCGGATGCAA for rat *ET-1*, Forward: CTTCCTGTGTTGTCCGCGCC, Reverse: AGGCCTCGTTGGCTGTCCTG for rat *LIMK2*. The reaction procedure was set as one cycle of 95 °C for 3 min, 40 cycles of 60 °C for 45 s and 95 °C for 10 s. GAPDH (Forward ACATCAAGAAGGTGGTGAAG; Reverse ATACCAGGAAATGAGCTTCA) was used as normalization for qRT-PCR data. Specificity of the PCR reactions was assessed by analysis of melting curves for each data point [42].

### Data analysis

Student *t*-test or one-way ANOVA was applied for statistical analyses. For post-hoc comparisons, we applied Bonferroni’s test. A *p*-value < 0.05 was considered statistically significant [52, 53].

## Abbreviations

CDK4: cyclin dependent kinase 4
DAPI: 4′,6-diamidino-2-phenylindol
DRP1: dynamin-related protein 1
DTT: dithiothreitol
ELISA: enzyme-linked immunosorbent assay
ET-1: endothelin-1
FJB: fluoro-Jade B
IF: Immunofluorescence
RIP1: receptor interacting protein kinase 1
ROCK: rho kinase
LIMK2: lim kinase 2
PBS: phosphate-buffered saline
SD: Sprague–Dawley
SE: status epilepticus
WB: Western blot
